# Parallel Exploitation of Diverse Host Nutrients Enhances *Salmonella* Virulence

**DOI:** 10.1371/journal.ppat.1003301

**Published:** 2013-04-25

**Authors:** Benjamin Steeb, Beatrice Claudi, Neil A. Burton, Petra Tienz, Alexander Schmidt, Hesso Farhan, Alain Mazé, Dirk Bumann

**Affiliations:** 1 Focal Area Infection Biology, Biozentrum, University of Basel, Basel, Switzerland; 2 Proteomics Core Facility, Biozentrum, University of Basel, Basel, Switzerland; Faculté de Médecine Paris Descartes, France

## Abstract

Pathogen access to host nutrients in infected tissues is fundamental for pathogen growth and virulence, disease progression, and infection control. However, our understanding of this crucial process is still rather limited because of experimental and conceptual challenges. Here, we used proteomics, microbial genetics, competitive infections, and computational approaches to obtain a comprehensive overview of *Salmonella* nutrition and growth in a mouse typhoid fever model. The data revealed that *Salmonella* accessed an unexpectedly diverse set of at least 31 different host nutrients in infected tissues but the individual nutrients were available in only scarce amounts. *Salmonella* adapted to this situation by expressing versatile catabolic pathways to simultaneously exploit multiple host nutrients. A genome-scale computational model of *Salmonella* in vivo metabolism based on these data was fully consistent with independent large-scale experimental data on *Salmonella* enzyme quantities, and correctly predicted 92% of 738 reported experimental mutant virulence phenotypes, suggesting that our analysis provided a comprehensive overview of host nutrient supply, *Salmonella* metabolism, and *Salmonella* growth during infection. Comparison of metabolic networks of other pathogens suggested that complex host/pathogen nutritional interfaces are a common feature underlying many infectious diseases.

## Author Summary

Infectious diseases are a major health problem worldwide. To cause disease, pathogens need to acquire host nutrients for growth in infected tissues and for the expression of virulence factors. In this study, we investigated *Salmonella* nutrition and growth in a well-characterized mouse model of human typhoid fever. We used a panel of *Salmonella* mutants with metabolic defects to assess the importance of various nutrient utilization pathways for *Salmonella* growth. We derived from these experimental data a computational model that predicts nutrient uptake rates, activity of metabolic pathways, and the effects of *Salmonella* enzyme defects on in vivo growth. The vast majority of these predictions were in close agreement with independent experimental data suggesting the model provided a consistent overview of *Salmonella* metabolism during infection. The data showed that *Salmonella* depend on a highly complex diet with many different host nutrients, but each of these nutrients is available in only scarce amounts. To grow and cause disease, *Salmonella* must simultaneously exploit these various nutrients with versatile degradation pathways. Similar complex pathogen diets might also drive many other infectious diseases.

## Introduction

Infectious diseases are a major worldwide threat to human health [1]. The situation is worsening because of rapidly rising antimicrobial resistance and insufficient development of new antibiotics. Most infectious diseases start with a few pathogenic organisms that invade host tissues, but disease symptoms develop only later when pathogens exploit host nutrients to grow to high tissue loads. Despite this crucial role of pathogen nutrition and growth, only a few host nutrients that are relevant for some pathogens have been identified [2], [3], [4], [5], [6], [7], [8], [9], [10], [11], [12], [13], [14], [15], and comprehensive quantitative data are lacking. The poor understanding of in vivo growth conditions can cause major antimicrobial drug development failures [16], [17], [18], [19] and might compromise antibiotic treatment [20].

In this study, we investigated *Salmonella* nutrition and growth in a mouse infection model mimicking human enteric fever. Enteric fever is caused by ingestion of food or water contaminated with *Salmonella enterica* serovars Typhi and Paratyphi (“typhoid/paratyphoid fever”) [21]. *Salmonella* invade intestinal tissues and disseminate to inner organs including spleen, liver, kidney, bone marrow, and brain, where they proliferate and cause tissue damages that can result in strong inflammation and organ failure. Enteric fever causes tremendous morbidity and mortality worldwide. Current control strategies become increasingly inefficient as a result of increasing antimicrobial resistance [22], [23] and emergence of *Salmonella* serovars that are not covered by currently available safe vaccines [24], [25].

In mice, *Salmonella enterica* serovars that cause human enteric fever usually do not cause any disease [26], in part because of expression of Toll-like receptor 11 in mice but not humans [27]. However, serovar Typhimurium, which can cause human diarrhea, causes in mice a systemic infection with pathology and disease progression similar to human typhoid fever. Some mouse strains carry a functional allele *Slc11a1* (also called *NRAMP*) coding for a Fe^2+^/Mn^2+^/Zn^2+^transporter, and such mice can successfully control systemic salmonellosis [28]. However, widely used laboratory mouse strains (i.e., BALB/c, C57BL/6) carry a dysfunctional *Slc11a1* allele which makes them highly susceptible to lethal systemic *Salmonella* infections. *Salmonella* infections in these genetically susceptible mice thus represent an excellent model for severe human typhoid (and paratyphoid) fever [26]. This disease model is particularly suitable for comprehensive experimental and computational analysis because of facile *Salmonella* genetics, availability of genome-scale in silico metabolic reconstructions [29], [30], [31], extensive literature, and close similarities between *Salmonella* and the prime model organism *E. coli*.

In this study, we used proteomics, mutant phenotyping, and computational approaches to investigate *Salmonella* nutrition and growth in this mouse typhoid fever model. Our data revealed an unexpectedly complex *Salmonella* nutritional landscape in infected host tissues, where many chemically diverse nutrients were available in scarce amounts. *Salmonella* adapted to this situation by simultaneously employing versatile nutrient utilization pathways.

## Results

### Extensive *Salmonella* nutrient utilization capabilities during infection

To characterize *Salmonella* metabolic capabilities during infection, we sorted *Salmonella* from infected mouse spleen and determined copy numbers of 477 metabolic enzymes (among 1182 identified proteins) using the well-established proteomics iBAQ label-free quantification approach [32] with 30 isotope labeled AQUA [33] peptides as internal standards (Table S1). This analysis extended our previous qualitative detection of 178 *Salmonella* enzymes in the same disease model [34] as a consequence of improved sorting and proteomics technologies.

**Figure 1:**
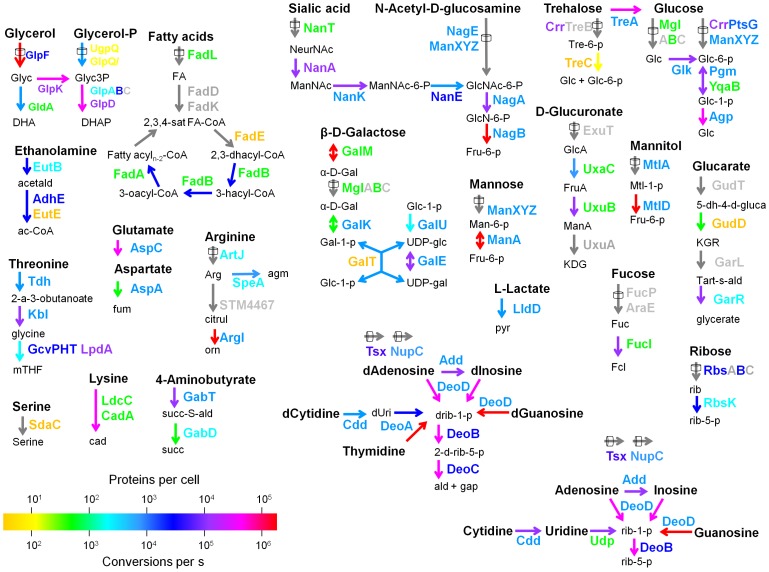
Nutrient utilization capabilities of *Salmonella* in infected mouse tissues. Colored names represent transporters and enzymes that were detected in *Salmonella* purified from mouse spleen (Table S1). The color shows enzyme abundance in copies per *Salmonella* cell. Grey proteins were not detected. Arrows represent metabolic reactions. Transport reactions are labeled with cylinders. Arrow colors show maximal catalytic capacities calculated from enzyme abundance and reported turnover numbers (Table S2). Grey arrows represent reactions, for which enzymes were not detected and/or turnover numbers were unavailable. Tsx is an outer membrane general nucleoside channel; NupC is a high affinity transporter for all nucleosides except guanosine and deoxyguanosine. An interactive map with detailed description of all detected metabolic capabilities is available at http://www.biozentrum.unibas.ch/personal/bumann/steeb_et_al/index.html.

The detected enzymes are known to catalyze 925 metabolic reactions, a remarkably high proportion of all known/inferred 2023 *Salmonella* metabolic reactions for which catalyzing enzymes have been annotated [31]. Interestingly, this included 102 transporters and enzymes involved in 77 reactions in 24 pathways for utilization of various carbohydrates, lipids, nucleosides, and amino acids (Figure 1). It is important to note that these enzyme numbers likely underrepresented the entire *Salmonella* in vivo proteome as limited material availability and mass spectrometry detection thresholds likely prevented identification of weakly expressed enzymes. These data suggested that during infection, *Salmonella* mobilized a large part of their diverse metabolic capabilities. In comparison, closely related *E. coli* requires only 293 reactions for optimal growth in a minimal in vitro medium [35]. However, even under such well-defined conditions, *E. coli* expresses more than 200 apparently not required enzymes suggesting that enzyme expression alone is not indicative of metabolic relevance [36] (see below).

In addition to the qualitative identification of expressed *Salmonella* enzymes and associated metabolic reactions, our proteome data also provided quantitative data on *Salmonella* metabolic capabilities. We combined enzyme copy numbers with available turnover numbers to calculate maximal reaction rates for 469 reactions (Table S2). As an example, we detected 20’000±1000 copies per *Salmonella* cell of glycerol kinase GlpK that catalyzes MgATP-dependent phosphorylation of glycerol to yield sn-glycerol 3-phosphate. The closely related *E. coli* ortholog (95% amino acid identity) has v_max_ = 22 µmol min^−1^ mg^−1^ [37] equivalent to a turnover number of 21 s^−1^. Based on these data, a single *Salmonella* cell would have the catalytic capacity to phosphorylate up to 420’000 glycerol molecules s^−1^. Such results should be taken as approximate only since turnover numbers are usually determined for somewhat non-physiological in vitro conditions (e.g., glycerol kinase was assayed in low osmolarity buffer at 25°C). Moreover, these data were incomplete because of undetected enzymes with abundance below the proteomics detection threshold and missing kinetic data. Nevertheless, the data yielded an unprecedented large-scale overview of *Salmonella* catalytic capacities in an infected host tissue, and provided a unique quantitative basis for in-depth analysis of metabolic activities involved in *Salmonella* virulence (Figure S1; an interactive map with detailed descriptions is available at http://www.biozentrum.unibas.ch/personal/bumann/steeb_et_al/index.html).

As expected [38], central carbon metabolism had particularly high catalytic power in contrast to biosynthesis pathways for minor biomass components such as vitamins. Many nutrient utilization pathways had also substantial catalytic power with especially high values for glycerol utilization (Figure 1). Together, these data suggested that *Salmonella* maintained versatile catabolic capabilities for diverse nutrients during infection.

### Functional relevance of diverse host nutrients

To determine the actual relevance of specific nutrients for supporting *Salmonella* host tissue colonization, we inactivated defined utilization pathways. We preferentially deleted transporters to prevent high-affinity nutrient uptake instead of inactivating degradation enzymes that could result in accumulation of toxic upstream metabolites such as phosphorylated carbohydrates, which can cause pleiotropic effects [39], [40]. Some nutrients can permeate membranes without a dedicated transporter (glycerol, short-chain fatty acids, myo-inositol, ethanolamine). In these cases, we inactivated enzymes that were unlikely to cause toxic intermediate accumulation based on available literature [41], [42], [43], [44].

Utilization defects have previously been used in several studies, for example to determine the relevance of several carbohydrates for *E. coli* growth in the intestinal lumen [2]. As a potential caveat, an excess supply of alternative nutrients may mask specific utilization defects. Moreover, some mutations might cause polar effects on the expression of downstream genes. In most of our mutants, this would only affect genes coding for subunits in the same transporters or enzymes involved in the same degradation pathways as the inactivated gene (Table S3). However, it was still possible that some such polar effects influenced mutant phenotypes.

In a complementary second set of *Salmonella* mutants, we inactivated well-characterized biosynthesis pathways for essential biomass components. The resulting *Salmonella* auxotrophs were unable to grow unless the missing biomass components were provided externally (Table S4). Any growth of such mutants in infected spleen was, therefore, indicative of host supply of the respective supplement. Similar approaches have previously been used in various infection models.

**Figure 2:**
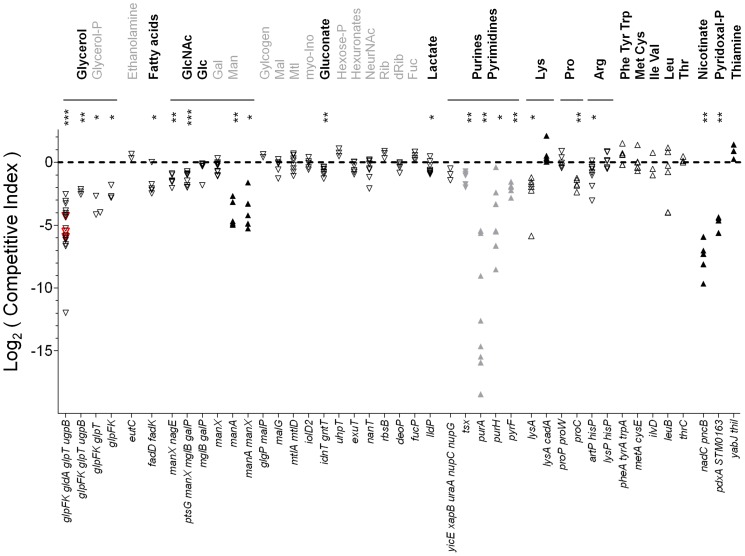
Mouse spleen colonization of *Salmonella* mutants with metabolic defects. The data represent competitive indices (CI) of mutants vs. wildtype *Salmonella* in spleen of individual mice at three (open symbols) or four days (filled symbols) post infection (Table S3). A log_2_(CI) value of 0 (equivalent to a CI of 1) represents full virulence. Down triangles represent mutants with utilization defects, up triangles represent auxotrophic mutants. Grey symbols represent data from a previous study [34] obtained in the same disease model. Red triangles represent data from an independently reconstructed *glpFK gldA glpT ugpB* mutant. The data provided evidence for access to a number of host nutrient which are shown in black (for detailed interpretation see Table S5). Nutrients with apparently low availability are shown in grey. Statistical analysis was carried out with the Benjamini-Hochberg false discovery rate (FDR) approach for multiple comparisons [45] (***, FDR<0.001; **, FDR<0.01; *, FDR<0.05).

To measure tissue colonization capabilities of the various mutants, we used competitive infections with mixtures of mutant and wildtype *Salmonella* (Figure 2; Table S3). Mutant fitness was measured as competitive indices (CI = output ratio (mutant/wildtype)/input ratio (mutant/wildtype)). A CI value of 1 (equivalent to log_2_(CI) = 0) indicated that a mutant had equal colonization capabilities as wildtype *Salmonella*. Complementation of mutant alleles to verify mutation phenotypes was often difficult because most strains contained multiple mutations. However, we independently reconstructed the most attenuated mutant and confirmed the resulting colonization defect (Figure 2). In the statistical analysis we avoided the “multiple comparison problem” using the widely accepted Benjamini-Hochberg [45] “false discovery rate” (FDR) approach to identify the subset of attenuated mutants (Table S3).

Interestingly, several *Salmonella* mutants with nutrient utilization defects had significantly diminished colonization capabilities (Figure 2; for detailed interpretation see Table S5). This suggested that there was no large excess of nutrients that would mask any utilization defects, and no single major nutrient that alone could support full *Salmonella* virulence. Instead, *Salmonella* colonization depended on utilization of glycerol, fatty acids, N-acetylglucosamine, gluconate, glucose, lactate, and arginine. Glucose was the only nutrient that had previously been identified to contribute to systemic *Salmonella* infection [11].

All seven identified nutrients can serve as a sole carbon source for *Salmonella* growth [46] and can be interconverted into each other. It was thus unlikely that any of these nutrients was required because it provided a unique chemical structure. Instead, the seven metabolites seemed to supply individual small nutritional contributions that only together enabled normal *Salmonella* in vivo growth (see below). Other utilization mutants had non-significant colonization phenotypes suggesting limited contributions of the corresponding nutrients.

Most of our infection experiments used BALB/c mice that carry a dysfunctional *Slc11a1* allele (see Introduction). Such mice are highly susceptible for systemic salmonellosis providing a useful model for severe human typhoid fever. For comparison, we also did some small-scale experiments in 129/Sv mice that carry a functional *Slc11a1* allele and are therefore resistant to lethal salmonellosis. Competitive infections confirmed the importance of glycerol (or glycerol-3-phosphate) and N-acetyl-glucosamine for *Salmonella* growth (Figure S2) suggesting similarities of *Salmonella* nutrition in susceptible and resistant mice.

Additional evidence for nutrient availability came from the substantial colonization capabilities of most tested *Salmonella* auxotrophs (Figure 2; Tables S3, S5). In particular, *Salmonella* readily accessed sufficient quantities of several (pro-)vitamins and all tested amino acids (except proline). Similar colonization phenotypes were obtained for *Salmonella* mutants with utilization or biosynthesis defects in infected liver (Table S3) indicating that similar nutrients supported *Salmonella* growth in two different host organs.

Combination of our data with previously reported additional mutant virulence phenotypes indicated *Salmonella* access to a large set of at least 31 chemically diverse host nutrients in infected mouse spleen (Table S5). This analysis thus revealed a highly complex host/*Salmonella* nutritional interface, which is still likely incomplete because of limited mutant coverage and our inability to detect small colonization defects.

### *Salmonella* virulence depends on parallel exploitation of diverse host nutrients

Our data suggested that *Salmonella* exploited a wide range of diverse host metabolites. This was initially surprising since most microorganisms utilize only a single preferred nutrient such as glucose when exposed to nutrient mixtures [47]. Other nutrients and their utilization pathways remain irrelevant as long as this preferred nutrient is available.

This was evidently not the case during infection, as glucose utilization only partially supported *Salmonella* growth in agreement with previous observations [11]. As one possible explanation, various nutrients including glucose might have been available in only limited amounts that together just supported *Salmonella* growth and tissue colonization. Indeed, colonization defects of *Salmonella* utilization mutants (Figure 2) suggested that *Salmonella* virulence depended on simultaneous effective exploitation of several nutrients instead of relying on only one preferred nutrient.

**Figure 3:**
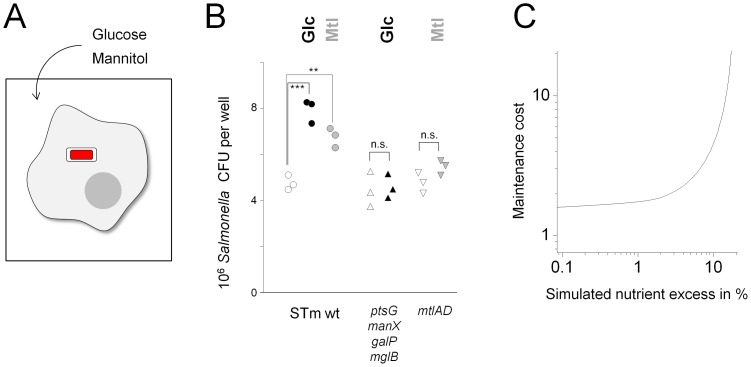
Nutrient limitation of intracellular *Salmonella* growth. **A**) Schematic representation of external supplementation of intracellular *Salmonella* (red) in infected macrophages (grey). **B**) Increasing external nutrient availability accelerates intracellular *Salmonella* growth, and this depends on specific *Salmonella* nutrient utilization capabilities (open symbols, 0.5 g l^−1^ glucose; filled black symbols, 1 g l^−1^ glucose; filled grey symbols, 0.5 g l^−1^ glucose 0.5 g l^−1^ mannitol; circles, wildtype *Salmonella*; upward triangles, *Salmonella ptsG manX galP mglB*, deficient for high-affinity glucose transport; downward triangles, *Salmonella mtlAD*, deficient for high-affinity mannitol transport and degradation). Colony-forming units (CFU) at 10 h post infection for triplicate wells containing 300’000 RAW 264.7 cells are shown. **C**) Flux-balance analysis of nutrient excess scenarios. The computational model was set to incorporate various amounts of excess nutrients (beyond what was needed for cell maintenance and growth). Model parameters were adjusted to yield predictions that were consistent with experimental mutant and wildtype colonization data. Simulation of up to 18% nutrient excess was possible but required unrealistically high maintenance costs (shown in multiples of maintenance costs for axenic conditions). Simulated scenarios with nutrient excess beyond 18% were incompatible with experimental colonization data.

To further test the hypothesis of parallel utilization of different available nutrients, we used a cell culture infection model where *Salmonella* replicated intracellularly in macrophage-like RAW 264.7 cells mimicking conditions during systemic salmonellosis [48]. In this cell culture model, extracellular metabolites can reach intracellular *Salmonella* and contribute to their nutrition [49], [50], [51], [52]. To test the impact of nutrient availability, we added external glucose or mannitol at 4 h post infection when *Salmonella* had already established their intracellular niche (Figure 3A). Interestingly, both extracellular nutrients accelerated subsequent intracellular *Salmonella* growth (Figure 3B). This growth promoting effect was dependent on specific *Salmonella* glucose/mannitol utilization capabilities, suggesting that external glucose and mannitol directly contributed to *Salmonella* growth, whereas nutrient-induced changes in the host cell had negligible impact (e.g., moderate changes in osmolarity (2.7 mOsm per added nutrient, some 1% of the total osmolarity), glucose metabolization by host cells (mannitol cannot be metabolized by mammalian cells [53]), or modulation of host cell phagocytosis and oxidative bursting as observed at much higher mannitol doses [54]). These data indicated that intracellular *Salmonella* growth was limited by nutrient availability, and *Salmonella* exploited both a typically preferred (glucose) and a non-preferred carbon source (mannitol) when available thus supporting our nutrient limitation hypothesis.

Taken together, both mutant colonization defects and cell culture experiments were consistent with *Salmonella* growth being dependent on diverse scarce nutrients during infection.

### Estimation of nutrient uptake rates

In addition to these qualitative results on nutrient-limited *Salmonella* growth, we were interested to obtain quantitative nutrient supply rates as a basis for comprehensive understanding and computational modeling of *Salmonella* nutrition, metabolism and growth. Quantitative nutrient supply rates have not yet been reported for any infection model, but the severity of mutant colonization defects could provide some hints. As an example, the strong colonization defect of *Salmonella glpFK gldA glpT ugpB* defective for glycerol utilization, compared to *Salmonella manX nagE* defective for GlcNAc utilization, could suggest that more glycerol was available as compared to GlcNAc. This rationale has previously been used to assess the relative relevance of various carbohydrates for *E. coli* gut colonization [2]. However, direct calculation of the respective nutrient supply rates from such mutant colonization defects was hampered by the parallel utilization of many diverse nutrients. Moreover, nutrients such as glycerol and GlcNAc differ in their nutritional value per molecule.

To quantitatively assess the availability of multiple host nutrients and their utilization by *Salmonella*, we therefore used a computational approach called Flux-Balance Analysis (FBA) [55]. This approach had been successfully applied to predict nutrient utilization and growth in a wide variety of organisms in excellent agreement with large-scale experimental data [56]. As a precondition for the application of FBA to *Salmonella*, we recently established together with more than 20 *Salmonella* experts an in silico reconstruction of the entire *Salmonella* metabolic network that contains all experimentally determined *Salmonella* metabolic activities, all enzymes with annotated metabolic activity encoded in the *Salmonella* genome, and their catalyzed reactions with all participating metabolites, stoichiometries, and information on reaction reversibility [31]. This consensus *Salmonella* metabolic reconstruction has been extensively documented and is continuously being updated by manual curation of newly available literature for *Salmonella* and closely related *E. coli* enzyme orthologs (reconstruction STMv1.1 with 1279 *Salmonella* enzymes, 1824 metabolites, 2573 reactions; Tables S6, S7, S8; the reconstruction is available in SBML format at http://www.biozentrum.unibas.ch/personal/bumann/steeb_et_al/index.html) and in the Supporting Information (Model S1).

Flux-balance analysis can be used to determine if the metabolic network is capable of producing all components required for *Salmonella* biomass generation. Importantly, biomass requirements can differ between growth conditions [57], [58]. To deduce *Salmonella* biomass requirements during infection, we analyzed published informative mutant virulence phenotypes and modified the biomass function accordingly (for detailed descriptions see Table S8; for limitations in the in vivo biomass definition see Discussion). Flux-balance analysis revealed that the metabolic reconstruction could generate all included biomass components in the correct stoichiometry under observance of fundamental thermodynamic laws such as preservation of mass and charge (“flux-balance”) [31]. In addition to biomass generation, all cells have growth-unrelated demands for survival and these are commonly accounted for as “maintenance requirements” [59]. Such demands could be especially important in pathogens during infection when they need resources to resist host antimicrobial defense.

To model *Salmonella* nutrition and growth in infected spleen, we provided the in silico reconstruction with all experimentally identified nutrients and used FBA to compute the resulting *Salmonella* biomass generation (which we used as an approximation for growth throughout this study). We adjusted nutrient uptake rates to reproduce our experimental *Salmonella* mutant phenotypes (for a detailed description of our approach, see Material and Methods and Figure S3). This yielded supply rates for 31 organic nutrients (Table S9), as well as 13 inorganic ions (Table S9). To obtain consistent data we needed to assume enhanced maintenance requirements (145±20% of the value for axenic in vitro cultures). Such enhanced maintenance costs could reflect defense against hostile host environments (see Discussion).

We also explored scenarios of excess nutrient supply (see Materials and Methods). The results revealed that the computation model could accommodate only modest nutrient excess up to 118% of the minimal nutrient supply values, and this would require improbably high maintenance costs for consistency with our experimental colonization data (Figure 3C). These data provided additional in silico support for nutrient-limited *Salmonella* growth (see above).

**Figure 4:**
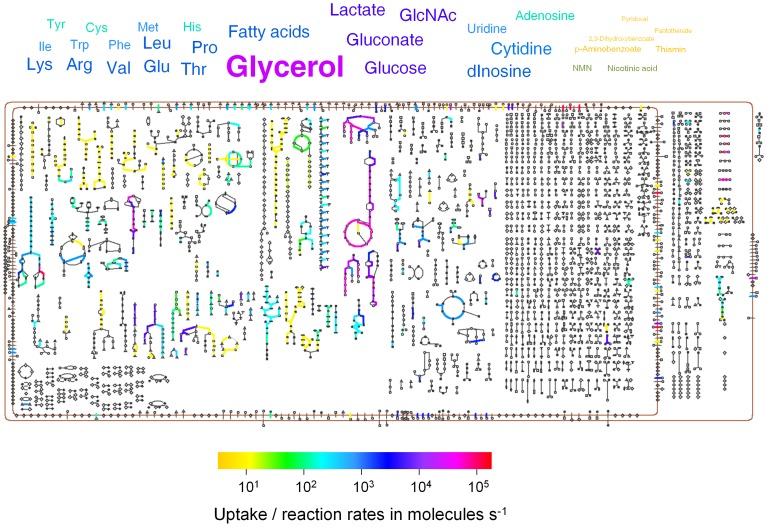
A quantitative genome-scale model of *Salmonella* nutrition, metabolism, and growth in infected mouse spleen. This schematic map shows available host nutrients, their respective uptake rates represented by color and font size, and their conversion to new *Salmonella* biomass through the *Salmonella* metabolic network (see text and Tables S6, S7, S8, S9 for detailed explanation and quantitative values). Symbols represent metabolites (squares, carbohydrates; pointing up triangles, amino acids; vertical ellipses, purines; horizontal ellipses, pyrimidines; pointing down triangles, cofactors; tees, tRNAs; circles, other metabolites; filled symbols, phosphorylated metabolites) and proteins (diamonds). The connecting lines present metabolic reactions. The brown lines represent the inner and outer membranes. An interactive map with detailed annotation of all reactions and the computational model in SBML format are available at http://www.biozentrum.unibas.ch/personal/bumann/steeb_et_al/index.html. The model is also available in the supporting information (Model S1).

It is important to note that our computational approach had several caveats (see Discussion). On the other hand, the resulting model provided a first comprehensive quantitative approximation to the host nutritional landscape and its exploitation by *Salmonella* that could serve as a basis for subsequent improvements (Figure 4; the model is available in SBML format at http://www.biozentrum.unibas.ch/personal/bumann/steeb_et_al/index.html) and in the Supporting Information (Model S1).

### Experimental validation of the model of *Salmonella* metabolism

To assess how well the current computational model captured relevant aspects of *Salmonella* nutrition and growth during infection, we compared model predictions with large-scale experimental data sets on *Salmonella* mutant phenotypes, enzyme expression, and metabolic capabilities.

**Figure 5:**
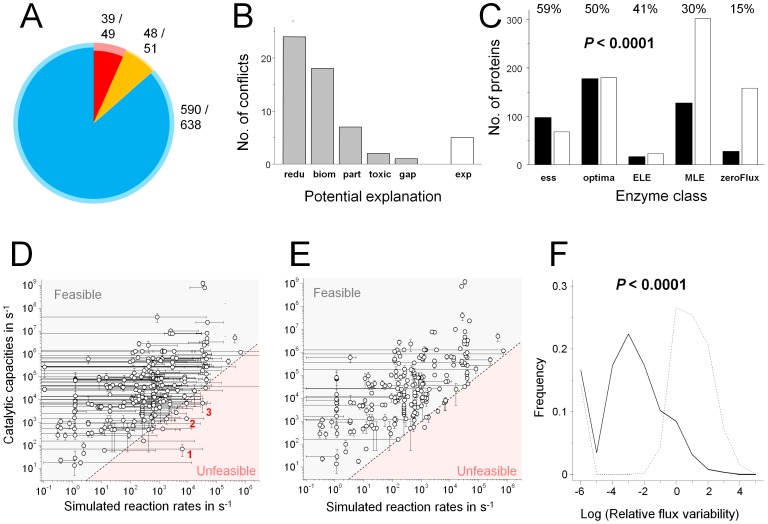
Large-scale experimental data are consistent with computational model predictions. **A**) Validation of mutant phenotype predictions. The colors show the predicted gene relevance for spleen colonization (red, essential; orange, contributing; blue, non-detectable; see text for definitions). Comparison of model predictions with 738 experimental *Salmonella* mutant phenotypes revealed 92% prediction accuracy (inner dark colors) but also 61 discrepancies (pale outer colors). Numbers (correct/total number of experimentally validated predictions) are also given. **B**) Potential reasons for inaccurate phenotype predictions (redu, unrealistic redundancy; biom, incomplete biomass/maintenance issues; part, partially contributing functions; toxic, accumulation of toxic upstream metabolites; gap, missing enzyme; or exp, possibly inaccurate experimental data). For detailed descriptions see Table S10. **C**) Detection of enzymes with predicted differential relevance for optimal *Salmonella* in vivo growth. Enzyme relevance was classified by parsimonious enzyme usage flux-balance analysis (pFBA) (ess, essential enzymes; optima, enzymes predicted to be used for optimal in vivo growth; ELE, enzymatically less efficient enzymes that will increase flux if used; MLE, metabolically less efficient enzymes that will impair growth rate if used; zeroFlux, enzymes that cannot be not used in vivo). Filled bars represent enzymes that were detected by *Salmonella* ex vivo proteomics, open bars represent enzymes that were not detected. Statistical significance of the relationship between enzyme classes and the proportion of detected proteins was determined using the Chi square trend test. **D**) Feasibility of predicted reaction rates. For each reaction, the range of flux rates compatible with full *Salmonella* growth was determined using Flux-Variability Analysis. The circles represent the most economical state with minimal total flux (see text). Predicted reaction rates are compared to corresponding catalytic capacities calculated form experimental enzyme abundance and turnover numbers (Table S2). The reddish area represents infeasible fluxes. Reactions with substantial infeasible fluxes in the most economic simulated state are labeled (1, formyltetrahydrofolate dehydrogenase; 2, phosphoserine aminotransferase; 3, glycerol dehydrogenase). **E**) Predicted flux ranges and corresponding catalytic capacities after constraining all reactions to feasible fluxes (except for the three aminoacyl tRNA ligations mentioned in the text). **F**) Relative flux ranges of the initial unrestrained (straight line) and the enzyme capacity-restrained (dotted line) models. For each reaction, the flux range was divided by the respective flux value in the most economical state. Reactions that carried no flux in the most economical state were not considered. Statistical significance of the difference between both distributions was tested using the Mann-Whitney U test.

To validate functional aspects of the computational model, we systematically predicted in vivo growth phenotypes for all 1279 model enzymes, and compared these predictions to reported experimental *Salmonella* mutant colonization phenotypes (Table S10; Figure 5A; interactive maps for predicted and experimental mutant phenotypes are available at http://www.biozentrum.unibas.ch/personal/bumann/steeb_et_al/index.html).

Inactivation of most enzymes had no impact on predicted growth rate. Only few, mostly biosynthetic, enzymes were essential for *Salmonella* virulence (predicted mutant growth rate below 60% of wildtype ), while some genes contributed to virulence (predicted mutant growth between 60% and 98% of wildtype), and the vast majority of enzymes had non-detectable effects (mutant growth rate higher than 98% of wildtype) in agreement with our previous experimental data [34]. Detailed analysis of 738 single mutants with available experimental data revealed an overall prediction accuracy of 92% (Figure 5A; Table S10) similar to accuracies achieved for the best computational models for *E. coli* in vitro cultures [59]. This analysis included 14 mutant phenotypes that we had used to deduce the biomass function (Table S8) and 69 mutation phenotypes that we had used to deduce nutrient supply (Table S5). Consistency of model predictions for these mutants and additional mutants with linked phenotypes (such as enzymes in the same pathways) was, therefore, unsurprising. Moreover, gene selection for mutant testing in our and other labs was likely influenced by previous knowledge. Mutant phenotypes thus did not provide truly independent validation, but they demonstrated that the model yielded consistent quantitative explanations for the function of hundreds of *Salmonella* genes during infection. On the other hand, there were 61 discrepancies between computational predictions and experimental data that could help to identify remaining errors and knowledge gaps (Figure 5B; for detailed analysis, see Table S10 and Discussion).

We also compared model predictions with our protein identification data. Specifically, we used a recently developed approach called parsimonious enzyme usage FBA (pFBA) [36] to predict enzyme classes with differential functional relevance for efficient *Salmonella* biomass generation. These enzyme classes included, in order of decreasing relevance, (i) essential enzymes, (ii) enzymes required for optimal growth with minimal overall flux (“optima”), (iii) enzymatically less efficient enzymes (which could sustain optimal growth but would require more total flux), (iv) metabolically less efficient enzymes (which could sustain only suboptimal growth), and (v) genes with no contribution to *Salmonella* growth (zero flux in the associated reactions). Comparison with our proteome data for ex vivo sorted *Salmonella* revealed that there was a statistically highly significant relationship between relevance and the proportion and abundance of detected *Salmonella* enzymes in the various classes (Figure 5C; Figure S4), similar to what has been observed for computational models of well-characterized *E. coli* in vitro cultures [36]. On the other hand, we still detected only some 50% of the relevant enzymes (classes “essential” and “optima”). Many non-detected enzymes were associated with rather low predicted reaction rates (Figure S5), suggesting that these enzymes might have been present in small quantities below our ex vivo proteome detection threshold. Incomplete proteome coverage of important enzymes has also been observed for *E. coli* in vitro cultures [36].

On the other hand, we detected several enzymes that were predicted to mediate no flux, again similar to observations for in vitro cultures [36]. Many such enzymes were involved in amino acid biosynthesis, nutrient utilization, gluconeogenesis, glycogen metabolism, and other pathways that all had experimentally non-detectable mutant phenotypes, consistent with their predicted non-functionality. It is possible, however, that these pathways were actually active, but accounted for minor contributions to *Salmonella* virulence that were undetectable with current in vivo methods. Alternatively, *Salmonella* might have prepared themselves for subsequent environments in their life cycle where these pathways would offer fitness benefits. Finally, *Salmonella* might be unable to optimally regulate its enzyme expression to shut down all dispensable enzymes (as it is likely the case in *E. coli* in vitro cultures). Further research is required to test these and other hypotheses.

We also compared our in vivo model with a model for *Salmonella* growth in minimal medium with glucose as sole source of carbon and energy. Interestingly, there was a large overlap between enzymes that were important for optimal growth of *Salmonella* under these two conditions. We detected 30 proteins that were predicted to be specifically required in vivo but not in vitro, providing some support for our in vivo model. On the other hand, we also detected 15 proteins that should be required only in the in vitro minimal medium but not in vivo. Interestingly, eleven of these 15 proteins were involved in amino acid biosynthesis suggesting that *Salmonella* maintained such biosynthetic capabilities in vivo despite access to host amino acids (see above). It is possible that the amino acid supply was just marginally sufficient and *Salmonella* prepared itself for future amino acid starvation. Further work is required to clarify this issue.

In addition to predicting enzyme relevance, the model also provided quantitative predictions for fluxes through hundreds of metabolic reactions. For some reactions, a large range of reaction rates was possible whereas others had more restricted rates (Figure 5D) as previously observed in other systems (“flux variability” [60]). We determined the state with the lowest overall metabolic activity corresponding to economical use of costly enzymes. Such states have shown to correspond well with experimental flux data in other systems [36], [61].

To determine the feasibility of these predicted reaction rates, we compared them to *Salmonella* catalytic capacities calculated from experimentally determined enzyme concentrations and turnover numbers (see above; Table S2; Figure S1; http://www.biozentrum.unibas.ch/personal/bumann/steeb_et_al/index.html). Interestingly, 459 out of 469 analyzed reactions had feasible predicted rates (Figure 5D). Three reactions had clearly infeasible reactions rates in the most economical computational state with lowest overall metabolic activity (>3 fold above the corresponding catalytic capacities; these reactions are labeled in Figure 5D: 1, formyltetrahydrofolate dehydrogenase; 2, phosphoserine aminotransferase; 3, glycerol dehydrogenase). However, all these reactions could be restrained to feasible rates without compromising predicted *Salmonella* growth or making other reactions infeasible. All seven other reactions had only moderate discrepancies between simulated and feasible rates, and four of them could again be restrained without compromising growth.

The remaining three reactions had simulated reaction rates that remained slightly infeasible in all states (simulated fluxes 1.2 to 2.5 fold too high). Interestingly, all three reactions were aminoacyl tRNA ligations (for proline, alanine, and threonine). Possible causes for these discrepancies included inaccurate biomass assumptions for proline, alanine, and threonine protein content, experimental errors in protein quantification, and/or suboptimal assay conditions for tRNA ligase turnover number measurements. Moreover, the computational model disregards important processes outside metabolism such as macromolecular expression [58], which could contribute to discrepancies between feasible and simulated reaction rates.

Despite these three minor discrepancies, the overwhelming feasibility of reaction rates indicated that *Salmonella* had sufficient in vivo enzyme amounts and catalytic power to mediate nutrient utilization, metabolization, and biomass generation as predicted by the computational model.

Although almost all reactions had entirely plausible reaction rates in the computational state with lowest overall metabolic activity, the entire flux solution space also included many reaction fluxes that exceeded plausible rates. In a next step, we prevented such implausible fluxes by setting upper/lower limits according to the maximum experimental enzyme capacities (except for the problematic three tRNA ligations, see above). Interestingly, these large-scale constraints still allowed normal *Salmonella* in silico growth, but resulted in a dramatically reduced flux solution space (Figure 5E, F). Specifically, the vast majority (80%) of reactions had narrowly defined flux ranges (relative flux variability below 10%), whereas in the initial unrestrained model only a small minority (16%) had such narrowly defined reaction rates (Figure 5F). This enzyme capacity-based model might thus provide a much better defined approximation to the actual in vivo flux state.

Together, these data revealed that the model predicted (i) largely correct mutant virulence phenotypes, (ii) predicted enzyme relevance that correlated with experimental protein detection, and (iii) predicted reaction rates that were biologically plausible. This large-scale consistency with experimental data suggested that the computational model captured major aspects of *Salmonella* nutrition, metabolism, and growth in infected host tissues.

### A common nutritional signature for mammalian pathogens

**Figure 6:**
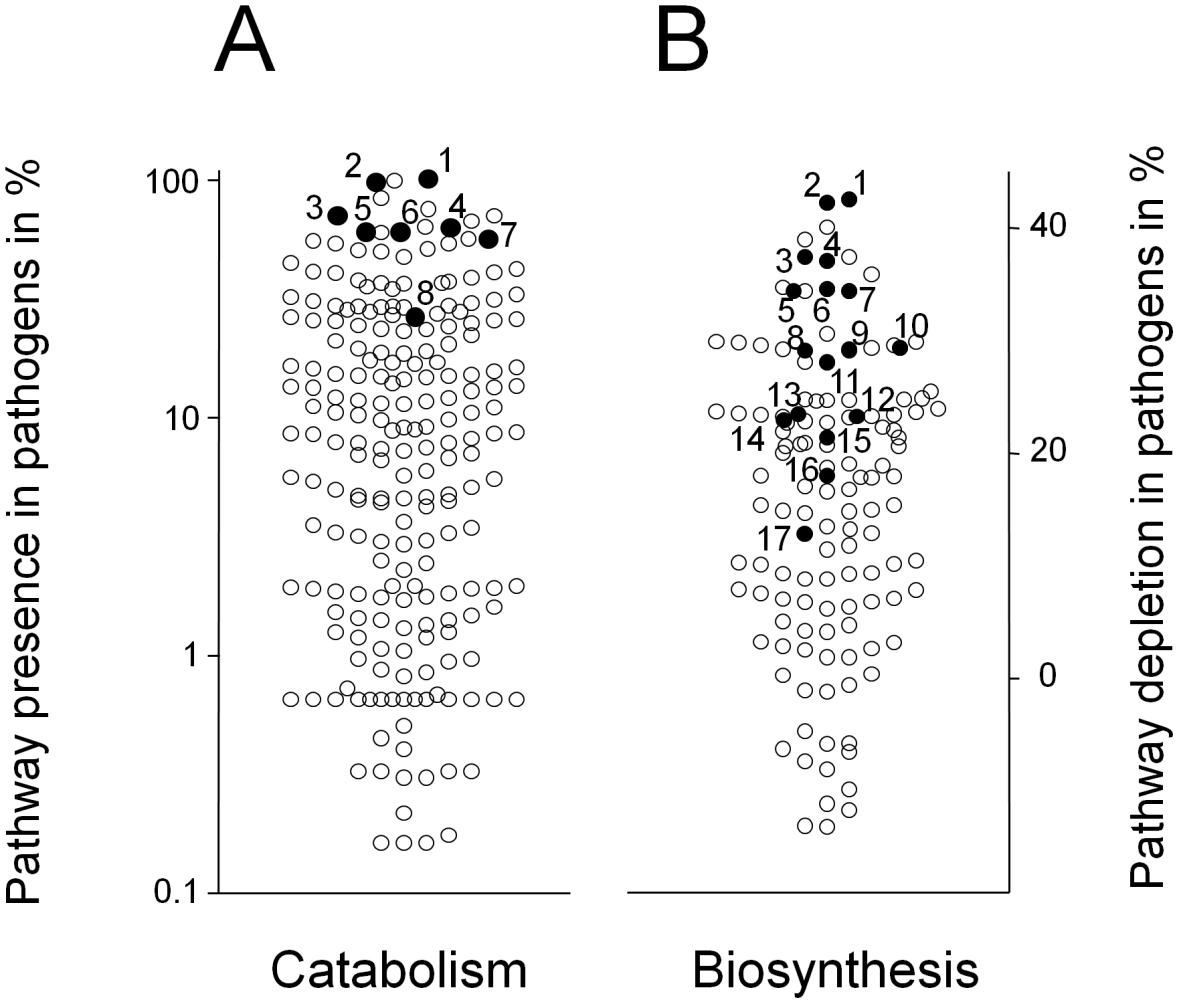
A common nutritional pattern for mammalian pathogens. **A**) Presence of 254 nutrient utilization pathways in genomes of 153 mammalian pathogens (excluding all *Salmonella* serovars). Data were based on pathway annotations available in MetaCyc [62]. Degradation pathways for nutrients that support *Salmonella* in mouse spleen were highly overrepresented among pathogen genomes (*P*<0.001; Mann-Whitney U test) suggesting similar nutritional preferences (filled circles; 1, purine nucleosides; 2, pyrimidine nucleosides; 3, fatty acids; 4, glycerol; 5, arginine; 6, N-Acetylglucosamine; 7, glucose; 8, gluconate). **B**) Depletion frequency of 118 biosynthesis pathways in mammalian pathogens. The values represent differences in pathway frequency in sets of 153 pathogens and 316 environmental bacteria (see text for explanation). Biosynthesis pathways for biomass components that *Salmonella* could obtain from the host were selectively depleted among pathogen genomes (*P*<0.0001; Mann-Whitney U test) suggesting similar host supplementation patterns (filled circles; 1, tyrosine; 2, histidine; 3, arginine; 4, cysteine; 5, methionine; 6, tryptophan; 7, threonine; 8, valine; 9 leucine; 10, isoleucine; 11, proline; 12, pyridoxal; 13, purine nucleosides; 14, pyrimidine nucleosides; 15, glutamine; 16, thiamin; 17, pantothenate).

To investigate if *Salmonella* conditions during mouse typhoid fever might be generally representative for pathogen nutrition in infected host tissues, we compared pathogen metabolic networks based on genome pathway annotations [62]. We analyzed 154 different mammalian pathogens (Table S11) for presence of 254 nutrient utilization pathways and 118 biosynthetic pathways (Figure 6A). Most pathogens shared the capability to utilize glycerol, fatty acids, various carbohydrates, nucleosides, and amino acids that could serve as N-sources (such as arginine), suggesting a general preference for nutrients that *Salmonella* used in the mouse typhoid fever model. Additional genome comparisons for 316 non-pathogenic species revealed that they might also preferentially utilize similar nutrients (Figure S6).

On the other hand, many pathogens have smaller genomes compared to related non-pathogenic species as a result of reductive genome evolution [63] resulting in loss of many pathways. To identify biosynthesis pathways that were commonly lost during this process, we determined a “biosynthesis depletion frequency” (DF) as follows. For each biomass component, we determined the fractions of pathogenic (P) and non-pathogenic (NP) species that encoded corresponding biosynthesis pathways in their genomes. The “biosynthesis depletion frequency” was calculated as the difference of the respective frequencies DF = NP−P. As an example, 89% of environmental bacteria but only 47% of mammalian pathogens had an apparently functional tyrosine biosynthesis pathway yielding a “depletion frequency” of 89%−47% = 42%. The results revealed that many pathogens lost biosynthesis pathways for several amino acids, nucleosides, and (pro)vitamins indicating that these pathogens - like *Salmonella* - might obtain such biomass components from their respective host environments (Fig. 6B).

Together, these genome comparisons suggested that many pathogens share with *Salmonella* access to a common large set of diverse host metabolites in infected mammalian tissues.

## Discussion

Host nutrients are essential for pathogen proliferation, disease progression, and efficacy of antimicrobial treatments. However, only few relevant nutrients have been identified and quantitative data on nutrient supply rates are lacking. In this study, we combined experimental enzyme abundance data, previously reported enzyme kinetic parameters, competitive infections with metabolic mutants, and computational modeling, to build and validate a comprehensive genome-scale model of *Salmonella* nutrition, metabolism, and growth in infected mouse tissues.

Virulence phenotypes of nutrient utilization mutants and auxotrophic mutants revealed that *Salmonella* accessed a surprisingly large number of chemically diverse host nutrients including lipids, carbohydrates, amino acids, nucleosides, and various (pro)vitamins. Surprisingly, this included facile availability of all three aromatic amino acids based on full virulence of auxotrophic *Salmonella pheA tyrA trpA*. This was also consistent with common tryptophan auxotrophy of *Salmonella* enterica serovar Typhi clinical isolates from human typhoid fever patients [64]. On the other hand, aromatic amino acids were previously thought to be unavailable in infected mouse tissues based on strongly attenuating mutations in chorismate biosynthesis [65], [66], a precursor for aromatic amino acids. However, such mutants are not informative for aromatic amino acid availability since chorismate is also a precursor for ubiquinone which is essential for *Salmonella* virulence [34]. Similar conditions might exist for intracellular *Listeria* [67].

The large diversity of accessible host nutrients posed complex challenges to *Salmonella* metabolism but, in addition, also relieved *Salmonella* dependence on any particular nutrient and its corresponding utilization pathway, thus enabling *Salmonella* to maintain high virulence even when biosynthesis pathways for important biomass components such as amino acids were defective. This buffering capacity of the complex nutritional landscape significantly contributed to the remarkable robustness of *Salmonella* metabolism against internal perturbations during infection [34].

Proteome analysis of *Salmonella* purified from infected tissues revealed in vivo expression of enzymes involved in degradation of the major nutrients glycerol, fatty acids, and N-acetylglucosamine, glucose, lactate, and arginine suggesting that *Salmonella* allocated major enzyme resources to relevant pathways in agreement with earlier observations [68]. Exceptions included mannose-6-phosphate isomerase (ManA) and UDP-glucose 4-epimerase (GalE) that can participate in degradation of mannose and galactose, respectively. Both enzymes were present in concentrations that would sustain high reaction rates (Figure 1), yet neither mannose nor galactose had a detectable nutritional contribution during infection (Figure 2; Table S5). However, both enzymes can also operate in reverse direction for biosynthesis, and corresponding mutant phenotypes [69], [70] support this as their dominant role in *Salmonella* virulence. Together, the proteome data revealed versatile *Salmonella* adaptation to a complex nutritional landscape.

To deduce quantitative in vivo supply rates for the various nutrients, we used a genome-scale computational approach based on *Salmonella* mutant colonization phenotypes. Specifically, we updated a genome-scale reconstruction of the *Salmonella* metabolic network and established a modified in vivo biomass composition. We then determined which nutrient uptake rates would support *Salmonella* biomass production consistent with experimental colonization data for wildtype and mutant *Salmonella*. This approach yielded uptake rates for 31 organic and 13 inorganic nutrients. For consistency with the experimental wildtype *Salmonella* in vivo generation time, we had to increase the non-growth associated ATP maintenance requirements to some 145% of their original value for axenic in vitro cultures [31]. Increased maintenance costs might be expected for hostile host environments compared to axenic in vitro cultures, but accurate experimental validation of maintenance requirements is generally challenging [59], [71].

It is important to note that our entire computational approach relied on several simplifying assumptions. (i) We disregarded nutrient utilization for purposes other than biomass generation or maintenance/virulence. (ii) We disregarded additional non-metabolic functions of the various mutated *Salmonella* genes (“moonlighting functions” [72]). Such additional functions are possible although they have not yet been observed for any of the specific transporters/enzymes that we had inactivated. (iii) We assumed similar in vitro and in vivo biomass composition (except for a few components for which informative mutant phenotypes had been reported) disregarding well-documented effects of differential growth rates on biomass composition [58], [73], [74]. (iv) We deduced average nutrient supply rates but conditions might change during infection and could also differ between various *Salmonella* subpopulations. Some kinetic information could be obtained from competitive infection time series but this would require an extensive number of experimental animals.

Because of all these caveats, the predicted nutrient supply rates and maintenance costs should be regarded only as rough estimates providing order-of-magnitude information as an approximation to the actual in vivo situation. On the other hand, the resulting model provided a first comprehensive quantitative approximation to the host nutritional landscape and its exploitation by *Salmonella* that could serve as a basis for subsequent improvements.

To assess how well the current stage of this model reflected *Salmonella* nutrition and metabolism during infection, we extensively validated model predictions with large-scale experimental data. Interestingly, enzymes with predicted high relevance for optimal *Salmonella* growth were experimentally detected at higher rates compared to non-functional enzymes. Moreover, rate predictions for hundreds of reactions were consistent with experimentally determined enzyme levels. This indicated that the simulated metabolic flux distribution was fully feasible with the amounts of enzymes that are actually present in *Salmonella* during infection.

The model predicted hundreds of mutant virulence phenotypes with an accuracy of over 90% thus indicating large-scale consistency with experimental data. The few remaining discrepancies may provide hints for further model improvements and targeted research to close knowledge gaps. Detailed examination suggested various typical limitations of our computational approach including (i) overestimated redundancies due to neglected regulation of isozyme/alternative pathway expression and/or differential substrate affinities (e.g., possibly poor expression of the sodium transporter NhaB which might fail to compensate for a *nhaA* defect in contrast to model predictions; low affinity zinc uptake through YgiE, which might be insufficient to compensate a defective ZnuABC zinc high-affinity transporter), (ii) incomplete biomass/maintenance functions that neglect signaling and detoxification needs (e.g., SpoT-dependent ppGpp homeostasis), (iii) inappropriate treatment of biomass components that contribute to virulence but are not absolutely essential (e.g., enterobacterial common antigen), (iv) neglect of continuous uptake of nutrients despite accumulation of toxic downstream intermediates (e.g., accumulation of GlcNAc-phosphate in absence of N-acetylglucosamine-6-phosphate deacetylase NagA [75]), and (v) knowledge gaps (e.g., bypass of dihydropteroate synthase FolP) (Figure 5B; for detailed analysis see Table S10). Subsequent model versions might overcome some of these limitations to further improve prediction accuracy. In addition, a few experimental data might possibly be wrong based on inconsistencies between different studies.

We also tested the predictive power of an enzyme capacity-restrained model that might more closely reproduce the wildtype flux state. Single gene deletion analysis of this model had very similar accuracy with three additional discrepancies (too severe predicted growth defects for *thrB*, *thrC*, and *aceA*) while resolving only one discrepancy (detectable growth defect of *zwf*) as compared to the unrestrained model. The enzyme capacities that we used as constraints in this model were based on protein profiles of wildtype *Salmonella*. Mutant *Salmonella* might have somewhat different protein profiles and enzyme capacities, and this might explain why the restrained model was not superior to the unrestrained model in predicting mutant colonization phenotypes.

Taken together, the excellent agreement of model predictions and large-scale experiment data suggested that the model accurately captured major aspects of *Salmonella* nutrition, metabolism, and growth during infection in a comprehensive and quantitatively consistent way.

Experimental mutant phenotypes and cell culture experiments suggested that despite *Salmonella* access to many host nutrients, these nutrients were available in only scarce amounts that individually would be insufficient to support full *Salmonella* virulence. This was also supported by modeling results that were incompatible with any substantial nutrient excess. *Salmonella* thus seemed to depend on simultaneous exploitation of several chemically diverse host nutrients through versatile utilization pathways. This apparent nutrient limitation inside infected host cells was initially surprising, since host cells contain numerous abundant metabolites that could provide rich carbon, nitrogen, and energy sources for *Salmonella*. However, intracellular *Salmonella* are separated from the nutrient-rich host cell cytosol by a vacuolar membrane that might restrict nutrient access. Further studies are required to better characterize this membrane and to test various hypotheses on host control of nutrient supply to *Salmonella*.

This study extends previous work on metabolic host/pathogen interactions. In particular, combinations of transcriptome data, mutant phenotypes, and genome-scale computational metabolism models have been used to analyze metabolism and growth of *Neisseria meningitidis* in serum [76], and *Mycobacterium tuberculosis* [57], [77] and *Listeria monocytogenes* [78] in macrophages. One study even incorporated the metabolic networks of both *Mycobacterium tuberculosis* and its infected host macrophage cell in one integrated model that describes the entire host/pathogen metabolic interaction [57]. These studies identified several relevant host nutrients such as amino acids driving pathogen growth and provided the first genome-scale descriptions of pathogen metabolism as a basis for a system-level understanding of metabolic host/pathogen interactions.

On the other hand, previous studies were limited to in vitro/cell culture conditions, included only a moderate number of host nutrients, and lacked quantitative data on nutrient supply rates and absolute enzyme levels. Our integrated experimental and computational approach addressed some of these limitations and yielded a comprehensive quantitative analysis of the highly complex nutritional in vivo landscape for *Salmonella* in infected host tissues. These data enabled us to generate a genome-scale model that accurately predicted enzyme requirements for *Salmonella* virulence in an important animal disease model.

However, there still remain important issues that should be addressed in future studies. (i) We interpreted net *Salmonella* colonization phenotypes always as division rate differences (similar to what has been done in most other studies). However, this is probably an oversimplification as some colonization defects might be caused by increased *Salmonella* killing by host antibacterial defenses, instead of differential *Salmonella* proliferation rates. In such cases, a simple metabolic interpretation in terms of diminished biomass production might be misleading. Future studies using methods such as Fluorescence Dilution [79] and direct detection of killed *Salmonella* [80] could provide suitable experimental data to address this issue. (ii) This and previous studies were based only on bulk measurements (transcriptomics, proteomics, mutant colonization phenotypes) that fail to account for any pathogen subpopulations. However, heterogeneous *Salmonella* subpopulations with different growth characteristics exist in vivo [79], [81]. So far, nothing is known about possible metabolic differences among distinct subpopulations, and future studies should address this issue since subpopulations might play important roles in virulence, transmission, persistence, and treatment failures [82]. (iii) A complete picture should include host metabolic processes that provide nutrients for *Salmonella*. An impressive study on tuberculosis already revealed some aspects of the interplay between host and pathogen metabolic networks in *Mycobacterium tuberculosis*-infected macrophages [57], and this approach might be extended to *Salmonella* as well. For *Salmonella* infections, analysis is complicated by the fact that the *Salmonella*-containing vacuole (SCV) communicates with late endosomes, from where it receives some incoming endocytosis cargo from the extracellular environment [51] thereby bypassing the metabolic network of the infected host cell. In addition, *Salmonella* might access some metabolites of the infected cell but additional experimental data will be needed to clarify the relative importance of the various nutrient supply routes. Another important aspect of metabolic host/*Salmonella* interactions is the question how *Salmonella* metabolism might influence host cell physiology. As an example, the capture by *Salmonella* of various host amino acids and nucleosides, as observed in this study, could modulate host cell functions that depend on these metabolites including antibacterial defense such as generation of nitric oxide [83]. Some indications for infection-induced changes in *Salmonella*-infected macrophages was already obtained in recent transcriptome and proteome studies [84], [85]. Increasingly accurate modeling of all these aspects might ultimately provide a complete quantitative description of the host/*Salmonella* metabolic interactions that enable *Salmonella* growth and enteric fever disease progression.

In addition to salmonellosis, the findings of this study also have some implications for infectious diseases in general. In particular, metabolic network comparisons suggested that many mammalian pathogens might share access to similar complex host nutrients that reflect general biochemical features of mammalian tissues. These results might provide a basis to establish in vitro culture conditions that closely mimic relevant in vivo conditions, helping to avoid drug development failures and to facilitate successful development of novel control strategies.

On the other hand, the actual relevance of individual nutrients can vary. As an example, ethanolamine is an important nutrient for *Salmonella* in inflamed intestine [13] but not in our systemic infections. As another example, *Mycobacterium tuberculosis* access fatty acids and proline (like *Salmonella* in mouse spleen), but glycerol is not a major nutrient, and lysine, tryptophan, and leucine are apparently available in insufficient amounts to meet mycobacterial biomass needs [19], [86], [87], [88], [89].

Interestingly, some of the commonly encountered nutrients are predominantly present as part of high molecular weight compounds such as glycans/glycoproteins (GlcNAc), proteins (most amino acids), or lipids (glycerol, fatty acids) suggesting that macromolecule hydrolysis might be an important aspect of pathogen nutrition in infected tissues. Indeed, many pathogens express hydrolases that degrade macromolecules such lipases, proteases, carbohydratases, etc., as part of their virulence program.

It might also be interesting to compare the common pathogen nutritional signature to the metabolism of commensal bacteria that inhabit body parts such as skin, genital mucosa, the oral cavity, or the intestine. Indeed, previous studies have already revealed commonalities among commensal gut bacteria such as the ability to digest complex carbohydrates [90]. Future studies might consider food components such as dietary plant sugars, host nutrients such as mucus, and waste products from other gut microbes. Moreover, such an analysis should also account for striking inter-individual differences in commensal microbial communities such as the recently described distinct enterotypes [91].

In conclusion, this study provided a comprehensive quantitative description of the *Salmonella* nutritional landscape during systemic salmonellosis and established a genome-scale model of *Salmonella* metabolism that explains major aspects of *Salmonella* infection biology. The results revealed an unexpectedly complex host/*Salmonella* nutritional interface that *Salmonella* exploited with versatile catabolic pathways. Similar complex host nutrients and versatile pathogen utilization pathways appear to be general features of many infectious diseases.

## Materials and Methods

### Ethics statement

All animal experiments were approved by Kantonales Veterinäramt Basel-Stadt (license 2239) and performed according to local guidelines (Tierschutz-Verordnung, Basel-Stadt) and the Swiss animal protection law (Tierschutz-Gesetz).

### Bacterial genetics

*Salmonella* mutants were constructed by lambda red-recombinase mediated allelic replacement [92] followed by general transduction using phage P22 *int* [93]. In multiple mutants, usage of the same resistance cassettes was enabled by FLP recombinase-mediated excision of the first cassette [92]. Strains were cultivated on Lennox LB medium containing 90 µg ml^−1^ streptomycin, 50 µg ml^−1^ kanamycin, 20 µg ml^−1^ chloramphenicol, and/or 100 µg ml^−1^ ampicillin. All auxotrophs required supplementation for growth as expected (Table S4).

### Mouse infections

We infected female, 8 to 12 weeks old BALB/c mice intravenously with 500–2000 CFU *Salmonella* from late exponential LB cultures. For some experiments, we used female, 8–12 weeks old 129/Sv mice. Three to four days post-infection (or five days for 129/Sv), mice were sacrificed and bacterial loads in spleen and liver were determined by plating of tissue homogenates treated with 0.3% Triton Tx-100. In competitive infections, wildtype and mutant *Salmonella* carrying different antibiotic resistance markers were mixed before administration. Individual strain tissue loads were determined by replica plating on selective media and competitive indices (CI = output ratio/input ratio) were calculated. Statistical significance was analyzed using t-test of log-transformed CI values (a parametric test was appropriate based on the normal distribution of such values [34]). Our experiments involved a large set of strains. To avoid the multiple comparison problem, we used the Benjamini-Hochberg false discovery rate (FDR) approach [45].

### Flow cytometry

For *Salmonella* ex vivo purification, *Salmonella sifB::gfp* [94] were sorted from infected mouse spleen as described [34] using a FACSAria III sorter (BD Biosciences). We used optical emission filters (green fluorescence, 499–529 nm; orange fluorescence, 564–606 nm) that optimally separated *Salmonella* GFP fluorescence from host cell autofluorescence. Proteome changes were minimized by preventing de novo synthesis with 170 µM chloramphenicol and delaying proteolysis by maintaining the samples at 0–4°C. Our previous results suggested that these conditions were effective to largely preserve the in vivo *Salmonella* proteome during sorting [34].

### Enzyme quantification using mass spectrometry-based proteomics

Preparation of tryptic peptides and analysis by LC-MS/MS was done essentially as described [95] with some modifications. Protein LoBind tubes and pipette tips (Axygen) were used throughout the procedure to minimize protein loss through adsorption. Frozen FACS sorted *Salmonella* pellets were resuspended in 15 µl lysis buffer (100 mM ammonium bicarbonate, 8 M urea, 0.1% RapiGest) and sonicated for 2× 30 seconds. Released proteins were reduced and alkylated, and first digested for 4 hrs with sequencing grade LysC peptidase (10 ng/µl; Promega) before overnight trypsin digestion. The detergent was cleaved by adding 2 M HCL and 5% TFA to final concentrations of 50 mM and 0.5% respectively, and incubating for 45 min at 37°C. Prior to centrifugation to remove the cleaved detergent (14,000×g, 10 min, 4°C), a mixture containing 32 heavy labeled reference peptides were added to the samples (5×10^−5^ fmoles per *Salmonella* for expected “high” abundance proteins, 5×10^−6^ fmoles per *Salmonella* for expected “low” abundance proteins; Table S12). The recovered peptides were desalted on C18 reverse-phase spin columns (Macrospin columns, Harvard apparatus), dried under vacuum and subjected to LC-MS/MS using an LTQ-Orbitrap-Velos instrument (Thermo-Fischer Scientific). Between 5×10^5^ and 2×10^6^ *Salmonella* sorted from individual mice were analyzed in replicate LC-MS/MS runs. In order to increase the number of *Salmonella* protein identifications, MS-sequencing was partially focused on previously identified *Salmonella* peptides using the recently developed inclusion list driven workflow [95]. Peptides and proteins were database searched against a decoy database consisting of the SL1344 genome sequence (ftp://ftp.sanger.ac.uk/pub/pathogens/Salmonella/), GFP, 204 frequently observed contaminants, all mouse entries from SwissProt (Version 57.12), and all sequences in reversed order (total 42502 entries) using the Mascot search algorithm. The search criteria were set as follows: full tryptic specificity was required (cleavage after lysine or arginine residues); 2 missed cleavages were allowed; carbamidomethylation (C) was set as fixed modification; oxidation (M) as variable modification. The mass tolerance was set to 10 ppm for precursor ions and 0.5 Da for fragment ions. The false discovery rate was set to 1% for protein and peptide identifications. In addition to *Salmonella* proteins a substantial number of contaminating mouse proteins were identified in the samples as previously noted [34]. Absolute quantities were determined for those 18–20 “anchor” *Salmonella* proteins that were detected along with a corresponding labeled AQUA peptide (Table S12) using the Trans-Proteomic Pipeline (TPP,V4.4.0). We then used the iBAQ method to establish absolute quantities of all remaining protein identifications, with a linear model error of between 47 and 60%. Comparison of samples from four independently infected mice revealed good reproducibility (Table S1). The data associated with this manuscript may be downloaded from ProteomeCommons.org Tranche using the following hash: HaSHrE4Paqa3Io3NARhJsV/7XeqYsNNvHYX3tt++xYcVYOf47nChKFB9E/PCD+j+xt5meJ1+4ytJIHVUeXx9Xb+ohBEAAAAAAAACZw =  = 

Enzyme abundance was combined with reported turnover numbers for the respective *Salmonella* enzymes (or closely related *E. coli* orthologs) to calculate maximal feasible reaction rates (Table S2). Data were visualized using the pathway tools package [96].

### Macrophage-like cell culture infection

Raw 264.7 macrophage-like cells were cultured in DMEM cell culture medium containing 10% serum and 0.5 g l^−1^ glucose. Cells were infected with *Salmonella* from stationary cultures at a multiplicity of infection of 30 for 30 min with an initial 5 min 1100×g centrifugation step. Medium was exchanged against DMEM containing 0.5 g l^−1^ glucose and 50 mg l^−1^ gentamycin. At 4 hours post infection, medium was exchanged with DMEM containing 0.5 g l^−1^ glucose, 1 g l^−1^ glucose, or 0.5 g l^−1^ glucose and 0.5 g l^−1^ mannitol. Cells were washed and lysed 10 h after infection, and aliquots were plated to determine CFU numbers.

### Computational modeling of *Salmonella* metabolism

The consensus genome-scale metabolism reconstruction STMv1 [31] was updated to STMv1.1 based on recent literature (Tables S6, S7). For in vivo modeling, we modified biomass requirements based on published mutant virulence phenotypes in infected host tissues. As an example, the high virulence of *Salmonella* mutants *rfbH*, *rfbJ*, *rfbV*, *rfbF*, *rfbG* [97] suggested that lipopolysaccharide with O-sidechains containing the carbohydrate abequose was not required in vivo. In total, these biomass modifications accounted for 14 mutant phenotypes (for detailed descriptions of all modifications see Table S8).

We generated an in vivo model using Flux-Balance Analysis (FBA) with the COBRA toolbox [98] in a MATLAB environment. Nutrient uptake rates were adjusted to yield consistent results with experimental competitive indices of *Salmonella* mutants and reported phenotypes (Tables S3, S5, S9) as well as the experimentally determined *Salmonella* wildtype in vivo generation time of 6.4 h [34] using the new MATLAB function nutrientUtilization() (Script S1). Specifically, uptake of each nutrient was varied and glucose was added to achieve the wildtype growth rate (in case of glucose as the nutrient of interest, we compensated with glycerol; in case of arginine that we modeled as a nitrogen source, we used ammonium for compensation). We then determined the nutrient uptake rate that matched the Competitive Index of a mutant that was unable to utilize this specific nutrient. After completing this procedure for each nutrient, we incorporated all first round nutrient uptake rates in an updated model. We then adjusted the maintenance costs to ensure a normal wildtype growth rate. We repeated this procedure a few times until values converged. We report these as final uptake rates in Table S9. We determined simulation error margins by analyzing error propagation from the experimental data (for examples, see Fig. S3). We used the calculated median uptake rate for 14 amino acids to estimate uptake of amino acids alanine, asparagine, aspartate, glutamate, glycine, and serine, for which we lacked informative mutant data. Biomass requirements suggested uptake rates for additional 13 inorganic components (Table S9).

We also explored the possibility of *Salmonella* access to excess nutrients using the new MATLAB function excess() (Script S2). Specifically, we increased the growth rate to higher values than experimentally observed. For these scenarios, we determined uptake rates for the six major carbon/energy sources and adjusted maintenance costs as described above. To calculate the corresponding nutrient excess, we then compared the total nutrient uptake for these scenarios to what would be needed for normal growth at the experimentally determined rate.

We predicted flux states with “minimal total flux” at maximal rates for biomass generation (“objective function”) using the respective options in the optimize() function. We determined flux variability in alternative solutions using the fluxVariability() function. This flux variability analysis was performed without assuming lowest overall metabolic activity to obtain the full range of possible flux states compatible with optimal *Salmonella* growth. We predicted biomass generation (which we used as an approximation for growth throughout this study) for all single gene deletions using the deleteModelGenes() function. Genes were defined as essential if predicted mutant growth rates were below 60% of wildtype (based on experimental growth data [34] for the avirulent *aroA* mutant [65]), contributing if mutants growth rates were between 60% and 98%, and non-detectable if mutants had growth rates higher than 98% of wildtype. We performed parsimonious FBA using the pFBA() function of the COBRA toolbox.

To validate these predictions, we examined reported experimental *Salmonella* colonization phenotypes and classified genes again as essential (lethal dose 1000fold higher than wildtype, or CI after four days below 0.005), contributing (significant colonization defect below thresholds for essential genes), or non-detectable (no significant difference to wildtype). We also used large-scale mutant phenotypes from two recent high-throughput studies [97], [99]. In these cases, we converted the reported mutant phenotype scores to growth rates and estimated confidence intervals based on the data provided (their Table S3 [97]; their Table S3 [99]) and the *Salmonella* in vivo generation time of 6.4 h in susceptible mice [34]. In cases where conflicting data had been reported, we preferentially used data from studies with low infection dose.

### Metabolic network comparisons

Metabolic Pathway predictions for 909 genomes were generated by the MetaCyc consortium [62] and kindly by provided Tomer Altman and Peter Karp on November 22, 2010. We identified 287 mammalian pathogens and 367 environmental organisms in this data set. We merged multiple strains belonging to the same species resulting in data for 154 pathogen species and 316 environmental species (Table S11). We then determined how many organisms in each group were capable to degrade a specific nutrient, or to synthesize a certain biomass component.

## Supporting Information

Figure S1 Metabolic capabilities of *Salmonella enterica* serovar Typhimurium in infected mouse spleen. Symbols represent metabolites (squares, carbohydrates; triangles, amino acids; circles, other metabolites; filled symbols, phosphorylated metabolites) and proteins (diamonds). The connecting lines present metabolic reactions. The brown lines represent the inner and outer membranes. Feasible reaction rates were calculated from in vivo enzyme abundance data and previously reported turnover numbers. An interactive version of this map with detailed descriptions for all reactions is available at http://www.biozentrum.unibas.ch/personal/bumann/steeb_et_al/index.html.
(TIF)

Figure S2 *Salmonella* mutant phenotypes in genetically resistant 129/Sv mice. Spleen colonization data are represented as competitive indices vs. wildtype *Salmonella*. A log_2_(CI) value of 0 (equivalent to a CI value of 1) indicates identical colonization of mutant and wildtype. Significance of attenuation was tested with t-test (*, *P*<0.05; **, *P*<0.01).
(TIF)

Figure S3 Determination of nutrient uptake rates which are consistent with corresponding mutant colonization phenotypes. Results for three mutants that were informative for access to proline, glycerol, and gluconate are shown.
(TIF)

Figure S4 Density plot of protein abundance for enzymes classified by parsimonious enzyme usage flux-balance analysis (pFBA) (ess, essential enzymes; optima, enzymes predicted to be used for optimal in vivo growth; ELE, enzymatically less efficient enzymes that will increase flux if used; MLE, metabolically less efficient enzymes that will impair growth rate if used; zeroFlux, enzymes that can not be not used in vivo). Abundance levels of undetected proteins were set to an arbitrary value of 10 copies per cell. Statistical significance of differences between essential enzymes and other classes was determined using the Mann-Whitney test.
(TIF)

Figure S5 Simulated rates for active reactions for which we detected the catalyzing enzyme(s) or not. The lines represent the medians. Statistical significance was determined using the Mann-Whitney test.
(TIF)

Figure S6 Presence of degradation pathways for various nutrients in pathogenic and non-pathogenic microbes. Nutrients that were shown to be utilized by *Salmonella* in infected mouse spleen are labeled with red crosses.
(TIF)

Table S1 Enzyme abundance in *Salmonella* sorted from infected mouse spleen as determined by quantitative proteomics.
(XLS)

Table S2 Feasible metabolic reaction rates in *Salmonella* during infection based on enzyme quantities and previously reported turnover numbers.
(XLS)

Table S3 Mouse spleen and liver colonization phenotypes of *Salmonella* mutants.
(XLS)

Table S4 In vitro growth characteristics of *Salmonella* auxotrophic mutants in chemically defined minimal M9 medium with or without supplementation.
(XLS)

Table S5 Evidence for *Salmonella* access to host nutrients based on mutant phenotypes.
(XLS)

Table S6 Novel metabolites included in an updated genome-scale *Salmonella* metabolic network reconstruction.
(XLS)

Table S7 Changed reactions in the *Salmonella* metabolic network reconstruction.
(XLS)

Table S8 Changed biomass components in the *Salmonella* metabolic network reconstruction.
(XLS)

Table S9 Simulated nutrient uptake rates and maintenance costs.
(XLS)

Table S10 Comparison of predicted and experimental mutant colonization phenotypes.
(XLS)

Table S11 Lists of pathogenic and environmental organisms included in metabolic network comparisons.
(XLS)

Table S12 Isotope labeled AQUA peptides for calibration of absolute enzyme quantities.
(XLS)

Model S1 Genome-scale metabolic in silico model of *Salmonella* in infected mouse spleen in SBML (Systems Biology Markup Language) format.
(TXT)

Script S1 MATLAB function for determination of nutrient uptake rates and maintenance costs from mutant colonization data.
(TXT)

Script S2 MATLAB function for determination of maintenance costs that are consistent with experimental data and nutrient excess scenarios.
(TXT)
