# Supplementary figures and images for: Parallel Exploitation of Diverse Host Nutrients Enhances Salmonella Virulence
Source: PLoS Pathog. 2013 Apr 25;9(4):e1003301. doi: 10.1371/journal.ppat.1003301 (PMC3636032; doi:10.1371/journal.ppat.1003301)

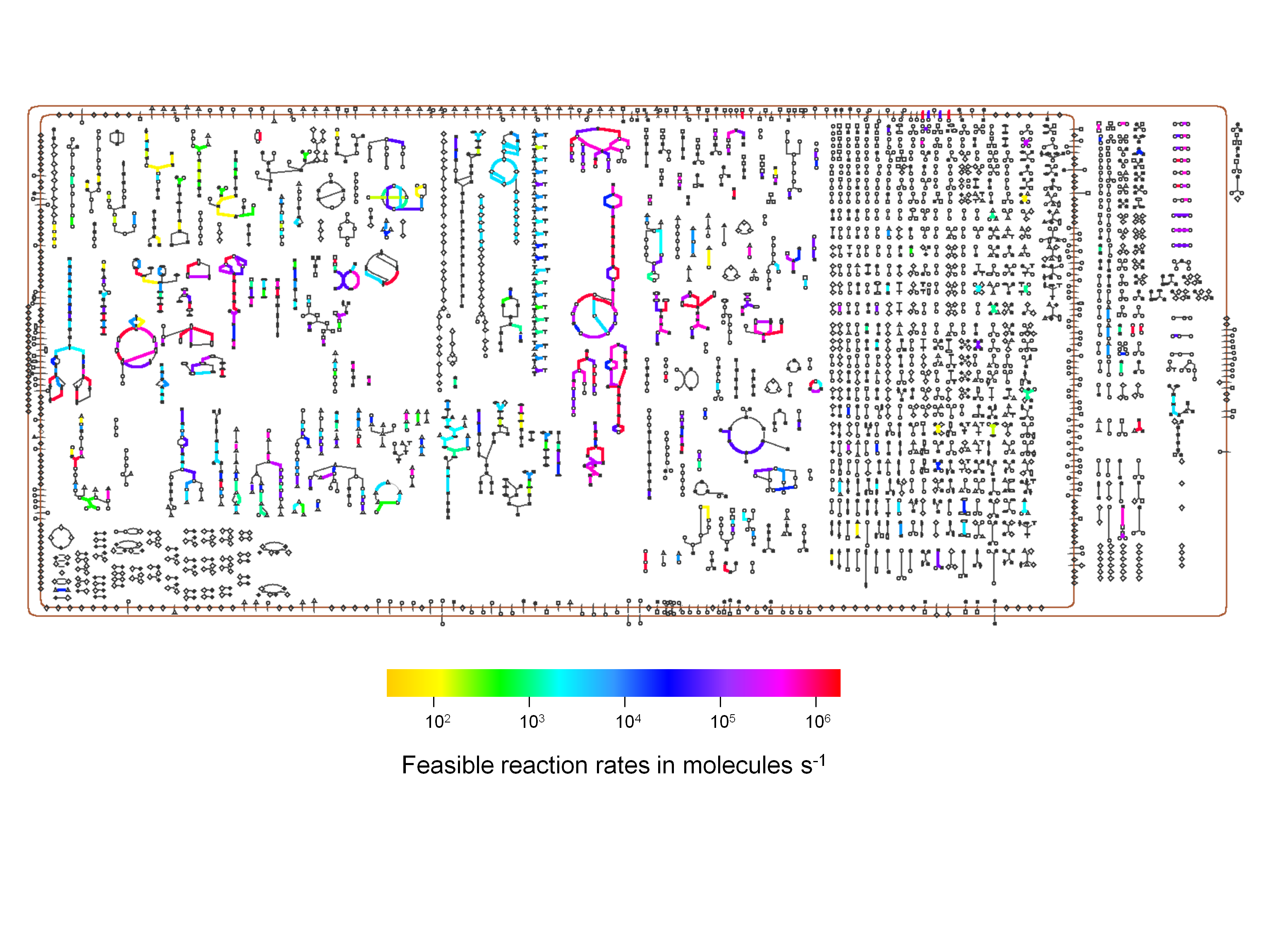

Supplement: Figure S1 — Metabolic capabilities of Salmonella enterica serovar Typhimurium in infected mouse spleen. Symbols represent metabolites (squares, carbohydrates; triangles, amino acids; circles, other metabolites; filled symbols, phosphorylated metabolites) and proteins (diamonds). The connecting lines present metabolic reactions. The brown lines represent the inner and outer membranes. Feasible reaction rates were calculated from in vivo enzyme abundance data and previously reported turnover numbers. An interactive version of this map with detailed descriptions for all reactions is available at http://www.biozentrum.unibas.ch/personal/bumann/steeb_et_al/index.html. (TIF) [file ppat.1003301.s001.tif]

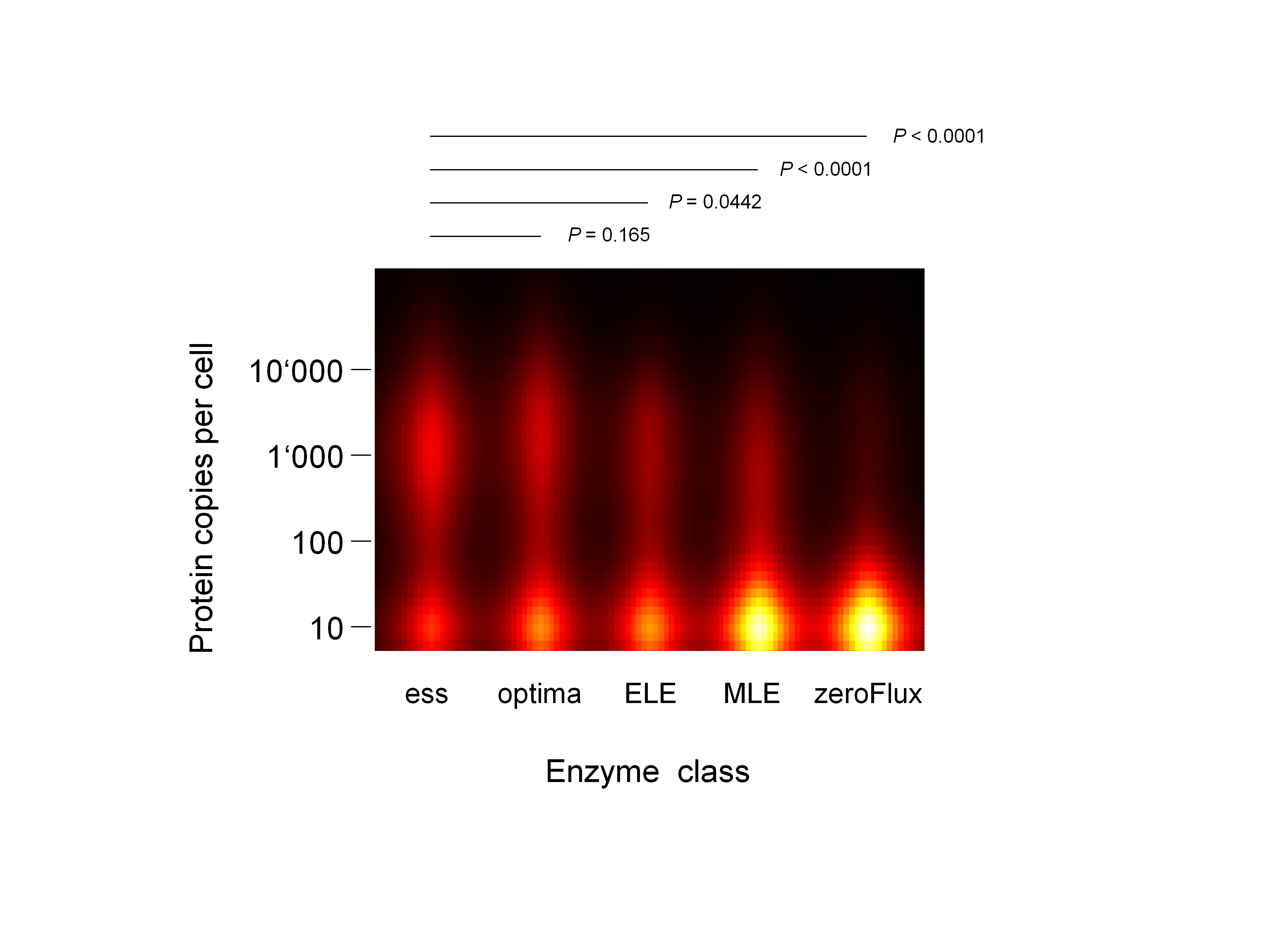

Supplement: Figure S4 — Density plot of protein abundance for enzymes classified by parsimonious enzyme usage flux-balance analysis (pFBA) (ess, essential enzymes; optima, enzymes predicted to be used for optimal in vivo growth; ELE, enzymatically less efficient enzymes that will increase flux if used; MLE, metabolically less efficient enzymes that will impair growth rate if used; zeroFlux, enzymes that can not be not used in vivo). Abundance levels of undetected proteins were set to an arbitrary value of 10 copies per cell. Statistical significance of differences between essential enzymes and other classes was determined using the Mann-Whitney test. (TIF) [file ppat.1003301.s004.tif]

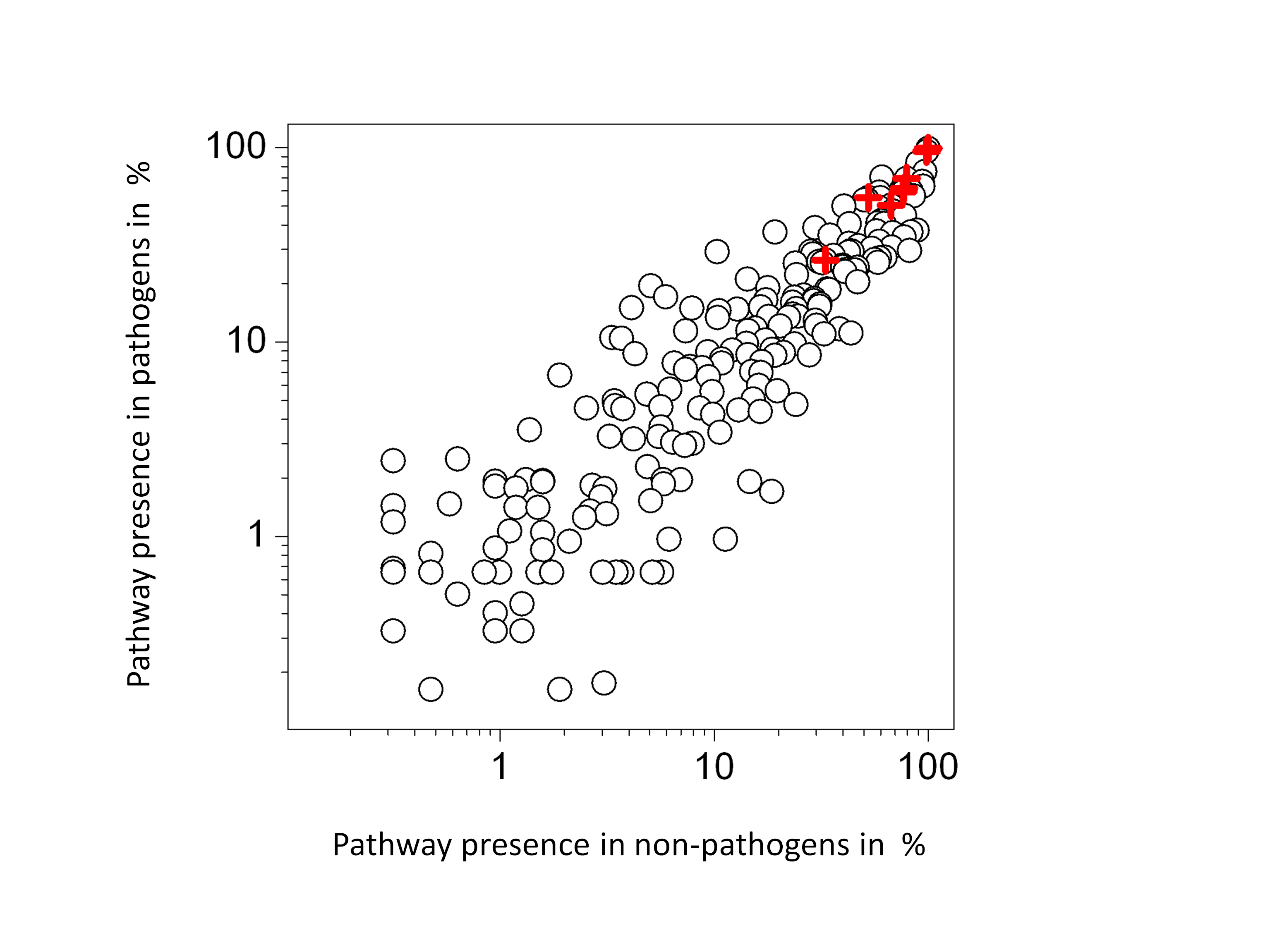

Supplement: Figure S6 — Presence of degradation pathways for various nutrients in pathogenic and non-pathogenic microbes. Nutrients that were shown to be utilized by Salmonella in infected mouse spleen are labeled with red crosses. (TIF) [file ppat.1003301.s006.tif]
